# Optimized amplitude modulated multiband RF pulse design

**DOI:** 10.1002/mrm.26610

**Published:** 2017-01-17

**Authors:** Samy Abo Seada, Anthony N. Price, Joseph V. Hajnal, Shaihan J. Malik

**Affiliations:** ^1^ Division of Imaging Sciences and Biomedical Engineering, King's College London London United Kingdom

**Keywords:** multiband pulse design, simultaneous multislice, root flipping, RF pulse design, excitation stability, interslice artifact

## Abstract

**Purpose:**

Multiband pulses are characterized by highly temporally modulated waveforms. Rapid phase or frequency modulation can be extremely demanding on the performance of radiofrequency (RF) pulse generation, which can lead to errors that can be avoided if pulses are restricted to amplitude modulation (AM) only. In this work, three existing multiband pulse design techniques are modified to produce AM waveforms.

**Theory and Methods:**

Multiband refocusing pulses were designed using phase‐optimization, time‐shifting, and root‐flipping. Each technique was constrained to produce AM pulses by exploiting conjugate symmetry in their respective frequency domain representations. Pulses were designed using the AM and unconstrained techniques for a range of multiband factors (ie, number of simultaneously excited slices), time‐bandwidth products, and slice separations. Performance was compared by examining the resulting effective pulse durations. Phantom and in vivo experiments were conducted for validation.

**Results:**

Acquired data confirmed that AM pulses can produce precise results when unconstrained designs may produce artifacts. The average duration of AM pulses is longer than the unconstrained versions. Averaged across a range of parameters, the duration cost for AM pulses was 26, 38, and 20% for phase‐optimizing, time‐shifting and root‐flipping, respectively.

**Conclusions:**

Amplitude modulation multiband pulses can be produced for a relatively small increase in pulse duration. Magn Reson Med 78:2185–2193, 2017. © 2017 The Authors Magnetic Resonance in Medicine published by Wiley Periodicals, Inc. on behalf of International Society for Magnetic Resonance in Medicine. This is an open access article under the terms of the Creative Commons Attribution License, which permits use, distribution and reproduction in any medium, provided the original work is properly cited.

## INTRODUCTION

Simultaneous multislice imaging 1, 2 can accelerate image acquisition, particularly for single‐shot imaging sequences used for diffusion and functional MRI. Simultaneous multislice imaging uses multiband radiofrequency (RF) pulses, which can be technically difficult to realize, especially for high acceleration factors. This is because the simple multiband pulse design method of time‐domain modulation leads to high‐peak RF power requirements (or correspondingly long durations). Recently, three methods have been proposed to tackle the peak power problem: phase‐optimizing 3, time‐shifting 4, and root‐flipping 5.

These three methods will usually produce complex‐valued RF pulses with rapid modulation of both amplitude and phase components. We observed that some clinical MRI systems struggle to recreate this rapid modulation. Specifically, we found that limitations in pulse generation when using an amplitude/frequency (AM/FM) representation lead to a rather noticeable degradation in performance, making use of this type of pulse problematic. We hypothesized that designing equivalent pulses that contain only amplitude modulation (ie, real‐valued waveforms) can effectively circumvent this issue. We present experimental results which demonstrate that this is indeed the case. In this work, we examine the three major multiband pulse design methods mentioned previously, demonstrate how each can be modified to produce AM‐only RF pulses, and show that in many cases the performance cost of doing so is modest. Hence, the use of AM‐only designs is a viable alternative for users experiencing this type of hardware issue.

## THEORY

The challenge of pulse design is to determine a RF magnetic field ( $B_{1}$) pulse that excites a desired transverse magnetization profile $M_{xy}$. The change in magnetization in the presence of $B_{1}$ and gradient fields is given by the Bloch equations, for which no analytic inversion exists with respect to $B_{1}$. One solution to this problem is to assume that the change in longitudinal magnetization is null. This is known as the small flip angle approximation, and simplifies the relation between $B_{1}$ pulses and resultant transverse magnetization to a Fourier relation 6. This is pertinent to multiband pulse design, as the simplest method for producing a multiband pulse b(t) is to take a single‐band pulse p(t) and multiply it by a modulation function f(t) as follows 1:

(1) $$b\left(t\right)=p\left(t\right)\overset{MB}{\underset{n=1}{\sum }}e^{i\omega_{n}t}=p\left(t\right)f\left(t\right)$$

Here, $\omega_{n}$ are the frequency offsets for the replica slices, numbered from n=1 to MB (multiband factor). This is referred to as in‐phase modulation, as all modulation functions are in phase with each other. The Fourier convolution theorem dictates that in the Fourier domain

(2) $$\tilde{b}\left(\omega \right)=\tilde{p}\left(\omega \right)*\tilde{f}\left(\omega \right)$$

where $\tilde{f}\left(\omega \right)$ is an array of delta functions centered on $\omega_{n}$. A key property is that conjugate symmetric Fourier series have real coefficients. The conjugate symmetric condition for a function x(t) with Fourier transform $\tilde{x}\left(\omega \right)$ is $\tilde{x}{\left(\omega \right)}^{*}=\tilde{x}\left(-\omega \right)$. Conjugate symmetric Fourier series have even magnitude functions and odd phase functions. In the following sections, we describe how each of the existing pulse design techniques can be modified to produce AM pulses.

### Phase‐Optimizing

Wong 3 showed that the peak of |f(t)| can be reduced by adding arbitrary phase offsets $\phi_{n}$, such that the overall modulation function is given by

(3) $$f\left(t\right)=\overset{MB}{\underset{n=1}{\sum }}e^{i(\omega_{n}t+\phi_{n})}$$

The peak amplitude of |f(t)| can be minimized by numerically optimizing the values of $\phi_{n}$. For the typical case in which $\omega_{n}$ are separated by a constant slice separation, distributed around $\omega_{0}$ (the center frequency of the slice group), the optimization can produce real‐valued multiband pulses if pairs of slices equidistant from $\omega_{0}$ have an antisymmetric phase offset as follows 7, 8:

(4) $$\begin{array}{c} \phi_{i}=-\phi_{j}\;\;\text{for}\;\;i,j\;\;\text{such}\;\text{that}\;\;\omega_{i}-\omega_{0}=\omega_{0}-\omega_{j}. \end{array}$$

**Figure 1:**
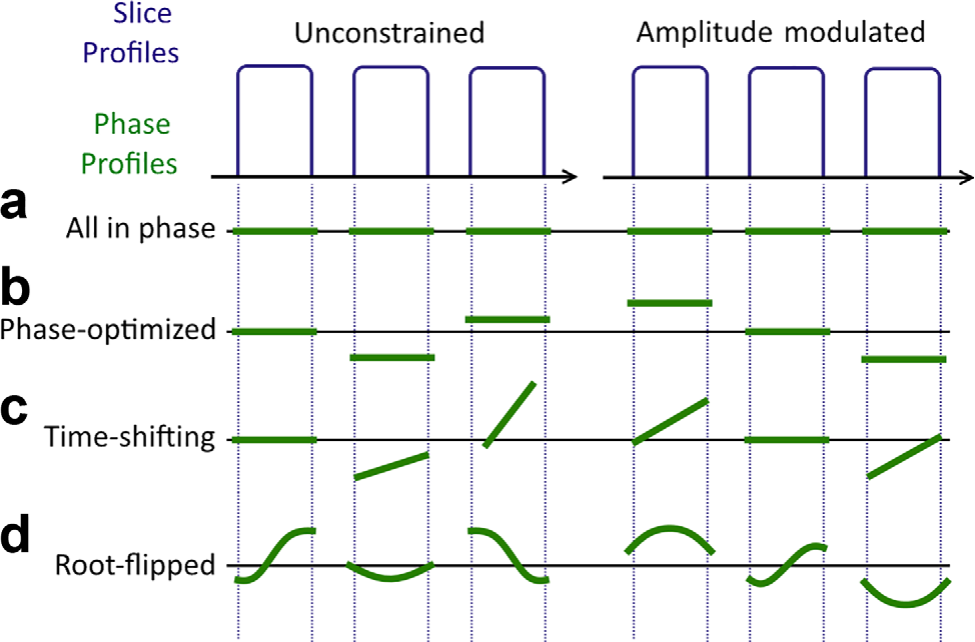
Illustration of multiband 3‐slice profiles for 90 ° excitation. The slice magnitude profiles are equivalent in all four methods: in phase, phase‐optimization, time‐shifting, and root‐flipping. The important difference between the unconstrained and amplitude modulated phase profiles is that the AM pulses result in antisymmetric phase profiles, such that the frequency response is conjugate symmetric.

When MB is odd valued, the central slice must be dealt with separately. Note that the optimal set of phase offsets for a multiband factor is independent of the single‐band pulse and the constant slice separation. The frequency behavior of in‐phase modulation and phase‐optimizing is illustrated in Figures 1a and 1b, respectively.

### Time‐Shifting

Auerbach et al 4 proposed an alternative to phase‐optimizing in which individual single‐band pulses are temporally offset to minimize constructive interference (with additional optimal phase offsets also calculated). It is no longer possible to separate the optimization design as the product between a single‐band waveform and a modulation function. Instead, the aggregated multiband pulse is characterized as

(5) $$b\left(t\right)=\overset{MB}{\underset{n=1}{\sum }}p\left(t-\tau_{n}\right)e^{i\left(\omega_{n}t+\phi_{n}\right)}$$

where $\tau_{n}$ is the temporal‐shift variable for each single‐band pulse. To create AM time‐shifted pulses, time‐shifting must be constrained such that single‐band pulses corresponding to slices equidistant from the center frequency are shifted in pairs, as follows:

(6) $$\tau_{i}=\tau_{j}\;\;\text{for}\;\;i,j\;\;\text{such}\;\text{that}\;\;\omega_{i}-\omega_{0}=\omega_{0}-\omega_{j}.$$

Similar to the phase‐optimizing method, when MB is odd, the middle slice must be dealt with separately. Because this method typically also includes optimized phase offsets, the condition from Equation 4 must also hold. When these conditions are met, the Fourier shift theorem implies that equidistant slices will gain similar linear phase ramps, allowing for conjugate symmetry to exist. This is in contrast to the original time‐shifting method, in which equidistant slices were effectively shifted in opposite directions in time. In contrast to phase‐optimizing, the optimal solution depends on the single‐band waveform p(t), so that optimal time and phase offsets must be evaluated for each design case. Figure 1c illustrates the relevant frequency behavior.

### Root‐Flipping

Root‐flipping, as proposed by Sharma et al 5, takes a direct approach to multiband pulse design using the Shinnar‐Le Roux (SLR) method 9. Briefly, this method involves designing a pair of polynomials of complex exponentials $\tilde{\alpha }$ and $\tilde{\beta }$, whose frequency representations yield the desired slice profile. Typically, the polynomial coefficients of $\tilde{\beta }$ are obtained using digital filter design; $\tilde{\alpha }$ can then be inferred using the equations 9

(7) $$\left|{\tilde{\alpha }}_{k}\right|=\sqrt{1-{\tilde{\beta }}_{k}{\tilde{\beta }}_{k}^{*}}=\sqrt{1-{\left|{\tilde{\beta }}_{k}\right|}^{2}}$$

(8) $$∠{\tilde{\alpha }}_{k}=H(\log\left|{\tilde{\alpha }}_{k}\right|)$$

(9) $${\tilde{\alpha }}_{k}=\left|{\tilde{\alpha }}_{k}\right|e^{i∠{\tilde{\alpha }}_{k}}$$

where H denotes the Hilbert transform. The time‐domain representations $\alpha_{n}$ and $\beta_{n}$ are then subjected to the inverse SLR transform to recover the required RF pulse b(t). ${\tilde{\beta }}_{k}$ can be expressed in polynomial form (as a sum of products) or in factored form (as a product of sums) as follows:

(10) β~k=∑n=0N−1βne−2πiknN=β0∏n=1N−1(1−rne−2πikN)

where $\beta_{n}$ are the polynomial coefficients, $r_{n}$ are the roots of the polynomial ${\tilde{\beta }}_{k},$ and  N is the degree of the polynomial.

When the roots are plotted on the complex plane, they are scattered around the unit circle. Flipping the passband roots radially from inside to outside or outside to inside the unit circle, changes the through‐slice phase profile without affecting the magnitude profile. In the time domain, root‐flipping redistributes the contributions associated with those frequencies across the duration of the RF pulse. The redistribution depends on the flipping pattern across the passband. Placing all roots inside (or outside) the unit circle leads to a maximum (or minimum) phase arrangement, and aligns the associated main amplitude peak at the start (or end) of the pulse. A search strategy can be used to find a flipping pattern that results in the distribution which leads to the minimum peak amplitude.

Amplitude modulated root‐flipped pulses can be designed by ensuring roots are located inside or outside the unit circle symmetrically about the real axis. This ensures that ${\tilde{\beta }}_{k}$ is conjugate symmetric, such that its time domain representation $\beta_{n}$ is real‐valued. When ${\tilde{\alpha }}_{k}$ is designed using Equations (7), (8), (9), in which $\left|{\tilde{\beta }}_{k}\right|$ is an even function, its magnitude function $\left|{\tilde{\alpha }}_{k}\right|$ will be even. Moreover, the phase response will be odd because the Hilbert transform of an even function is odd 10. Hence, ${\tilde{\alpha }}_{k}$ will be conjugate symmetric, and finally the inverse SLR transform 9 yields an AM RF pulse when both $\alpha_{n}$ and $\beta_{n}$ are real‐valued. An illustration for the conjugate symmetric condition for each of the three techniques is shown in Figure 1.

## METHODS

Pulses were designed for multiband factors 3 to 16, time‐bandwidth products (TBP) 2, 4, 6, 8 and 10, and slice separations of 3 to 22 slice thicknesses in integer steps. Thus, a total of 14  × 5 × 20=1400 cases were examined.

Single‐band SLR refocusing pulses were generated from filters designed using a modified version of the minimization proposed as Equation 1 in 5; the ripple limits on the minimization were modified to produce linear phase pulses, whereas in 5 the method was used to make minimum phase designs. Refocusing pulses were designed to produce matched excitation spin‐echo profiles with 1% ripples in and out of slice. These were used as a starting point for the phase‐optimization and time‐shifting results, to make them comparable with root‐flipping. Optimal phase offsets were found using the fmincon function in MATLAB (MathWorks, Natick, MA, USA) with 100 different random start points. Table 1 lists the optimized AM phase offsets for different multiband factors. For time‐shifting, multiband pulses were created by summing up uniformly time‐shifted single‐band pulses, as in 4. For the AM case, the shifts were still spaced linearly over the extended duration of the pulse, but with equidistant slices shifted in pairs as discussed previously. For each design case, 50 candidate time shifts were tested, with overall pulse durations ranging from 100% of the single‐band duration (no shift) to 200% (doubled duration). In each case, optimal phase offsets were found by using the genetic algorithm in MATLAB with population size 50, to then seed a local optimization with the fmincon function. Out of the 50 candidate solutions for each design case, the one with the lowest product between resulting pulse amplitude and duration was selected as optimal. The AM pulses were produced in the same way, except time shifts and phase offsets were applied in pairs, as described previously.

**Table 1:** Optimized Phase Offsets for AM Multiband Pulses

**MB**	Phase Offsets (deg)											
**3**	73.6	0	−73.6									
**4**	55.8	78.6	−78.6	−55.8								
**5**	66.3	−56.9	0	56.9	−66.3							
**6**	96.9	161.1	66.3	−66.3	−161.1	−96.9						
**7**	147.9	−32.2	5.9	0	−5.9	32.2	‐147.9					
**8**	121	12.6	83.9	114.1	−114.1	−83.9	−12.6	−121				
**9**	27.5	−152.8	−37	−24	0	24	37	152.8	−27.5			
**10**	96.4	−137.2	166.9	17.4	42.2	−42.2	−17.4	‐166.9	137.2	−96.4		
**11**	80.5	50.8	−106.2	4	−85.6	0	85.6	−4	106.2	−50.8	−80.5	
**12**	99.1	25.4	41.3	125.5	−125.8	56.4	−56.4	125.8	−125.5	−41.3	−25.4	−99.1

Note: Phases are ordered such that the central value corresponds to the middle slice.

Root‐flipped refocusing pulses were designed using code made available online by Sharma et al (www.vuiis.vanderbilt.edu/~grissowa/), again with 1% passband and stopband ripple constraints. The AM pulses were designed by modifying this code to flip roots as described previously. Half of the stopband roots at frequencies beyond the outermost passband were also flipped to prevent the accumulation of high coefficients at the pulse edges (Supporting Fig. S1). The Monte‐Carlo approach used by Sharma et al to find optimal root‐flipping patterns was replaced by the genetic algorithm toolbox in MATLAB, which we found to produce slightly improved results. For simplicity, we refer to the original root‐flipping method as “unconstrained,” even though it was constrained to be time‐symmetric.

In all three cases, optimal pulses were found independently for the proposed AM constrained and unconstrained cases. Because amplitudes and durations of RF pulses can be traded off, we have quantified the relative performance of each design by computing the “effective duration” $t_{eff}$, which we define as the duration relative to a hard pulse of equivalent peak amplitude and flip angle:

(11) $$t_{eff}=\frac{T\gamma \;b_{max}}{\theta }$$

where *T* is the pulse duration; $b_{max}$ is the maximum B_1_ amplitude; θ is the design flip angle; and γ is the gyromagnetic ratio. A doubling of $t_{eff}$ indicates a pulse that requires twice the amplitude for a given duration or vice versa. For comparison, the single‐band refocusing pulses used in this work had $t_{eff}\;$ = 3.637, 8.270, 13.231, 18.156, and 23.051 for TBP from 2, 4, 6, 8 and 10, respectively.

Other investigators 11 have found that pulses with a large degree of “roughness” in the RF envelope can lead to errors on some systems. Constraining pulses to be amplitude modulated could conceivably increase this roughness by removing the degrees of freedom associated with phase modulation. To investigate this effect, pulse envelope roughness was quantified using the measure suggested in 11 as follows:

(12) $$Roughness=\sqrt{\frac{1}{N-1}\overset{N-1}{\underset{n=1}{\sum }}{\left(\frac{\left|b_{n+1}\right|-|b_{n}|\;}{dt}\right)}^{2}}$$

where *N* is the number of time points used, and dt is the dwell time for which the pulse is evaluated.

The MATLAB code to reproduce all of the designs used in this work is available at https://github.com/mriphysics/AM_multiband/, including modifications made to the original root‐flipped design code, which was downloaded from www.vuiis.vanderbilt.edu/~grissowa/.

### Experiments

All experiments were conducted using a 3T Philips Achieva TX (Philips Healthcare, Best, the Netherlands) scanner with software release R3.2. This scanner uses analog spectrometer technology and requires RF pulses to be specified using an AM/FM representation.

**Figure 2:**
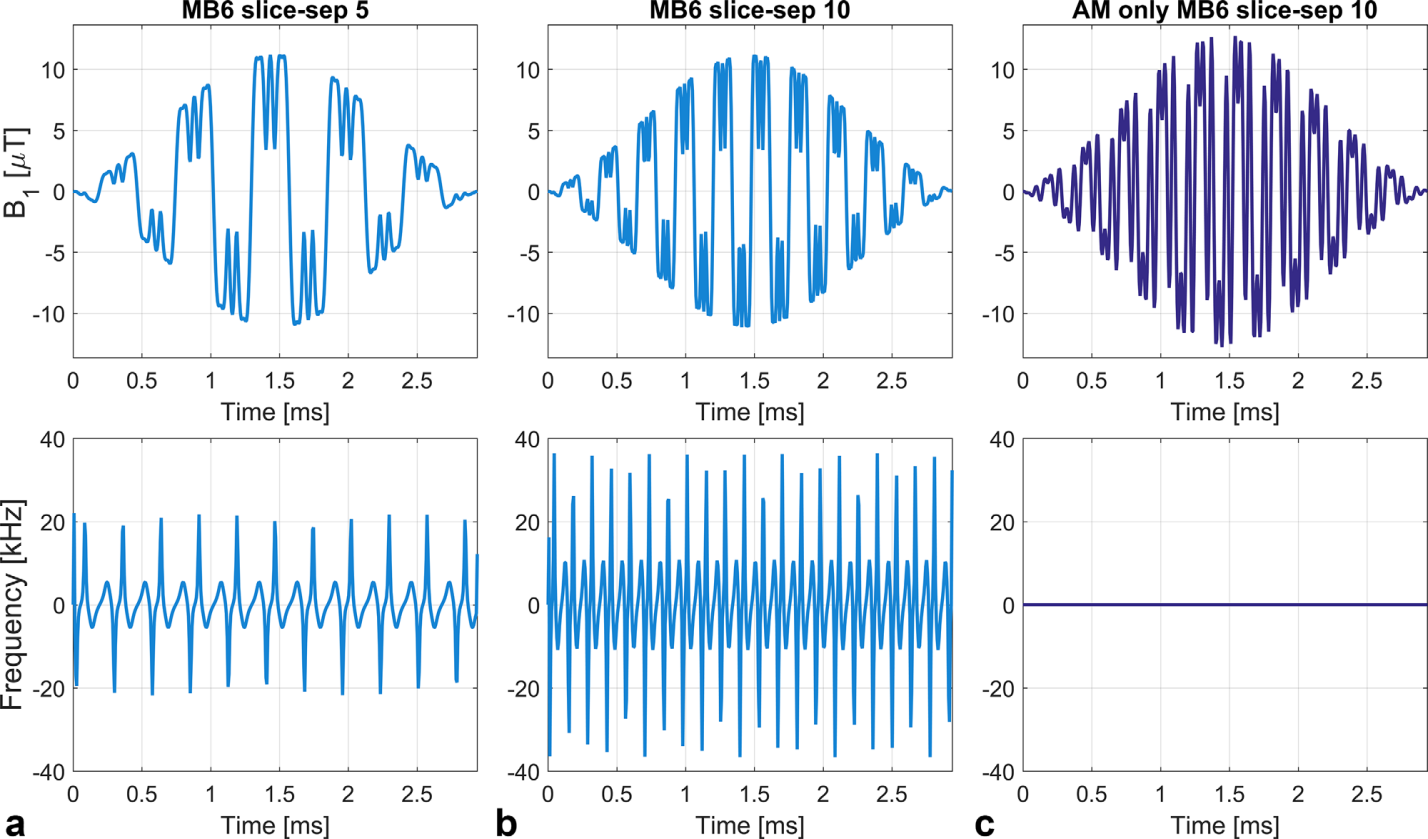
Sample multiband 6 waveforms designed using phase‐optimization, expressed as signed AM and FM. (**a, b**) Unconstrained phase‐optimized pulses. Doubling the slice separation from 5 slices to 10 increases the amplitude of the FM waveforms. (**c**) Using AM phase‐optimization, there is no FM. All three pulses are matched in duration. The AM pulse in (c) has a higher peak amplitude. The pulses in (b) and (c) have equivalent simulated slice profiles (eg, same magnitude profiles, ripple characteristics).

Slice‐profile measurements were performed on a long cylindrical phantom containing 100 mL saline doped with 1% gadolinium contrast agent (0.5 mmol/mL Gd‐DOTA, Dotarem, Guerbet LLC, Bloomington, IN, USA). For this experiment, phase‐optimized 90 ° multiband excitation pulses with 6 slices were designed based on a standard vendor pulse (TBP = 2.13) using both the AM and unconstrained approaches. Slice thickness was 1 mm and a range of slice gaps were included; increasing the slice gap leads to an increase in the size of frequency modulation, as shown by the examples in Figure 2. All pulses were designed at the scanner's RF dwell time (6.4 μs) to avoid additional artifacts from resampling; durations were matched at 2.94 ms and peak amplitudes were allowed to vary accordingly so that the flip angle remained constant. Slice profiles of the individual excitation pulses (ie, not spin echo pairs) were measured using a 2D gradient‐echo sequence with the read‐out gradient moved to the slice‐select direction. The acquired resolution was 0.1 mm through‐slice with repetition time (TR) = 100ms, echo time (TE) = 13 ms.

An in vivo imaging experiment was conducted with unconstrained and AM phase‐optimized versions of an MB4 pulse, for which the underlying single‐band waveform was a standard vendor pulse with TBP 3.05. A gradient echo single‐shot EPI sequence with blipped‐CAIPI acquisition scheme 12 (1 mm isotropic resolution, flip angle 52 º, TR 2 s, TE 25 ms) was used for acquisition with a 32‐channel head coil. Axial brain images (total coverage 120 mm) were acquired on a single healthy volunteer (written, informed consent obtained before enrollment), and data were reconstructed with a SENSE‐based algorithm using ReconFrame (GyroTools GmbH, Zurich, Switzerland). As with phantom experiments, the pulse duration was the same for the unconstrained and AM pulses (2.39 ms), so that the bandwidth properties were the same in both acquisitions.

## RESULTS

### Simulations

**Figure 3:**
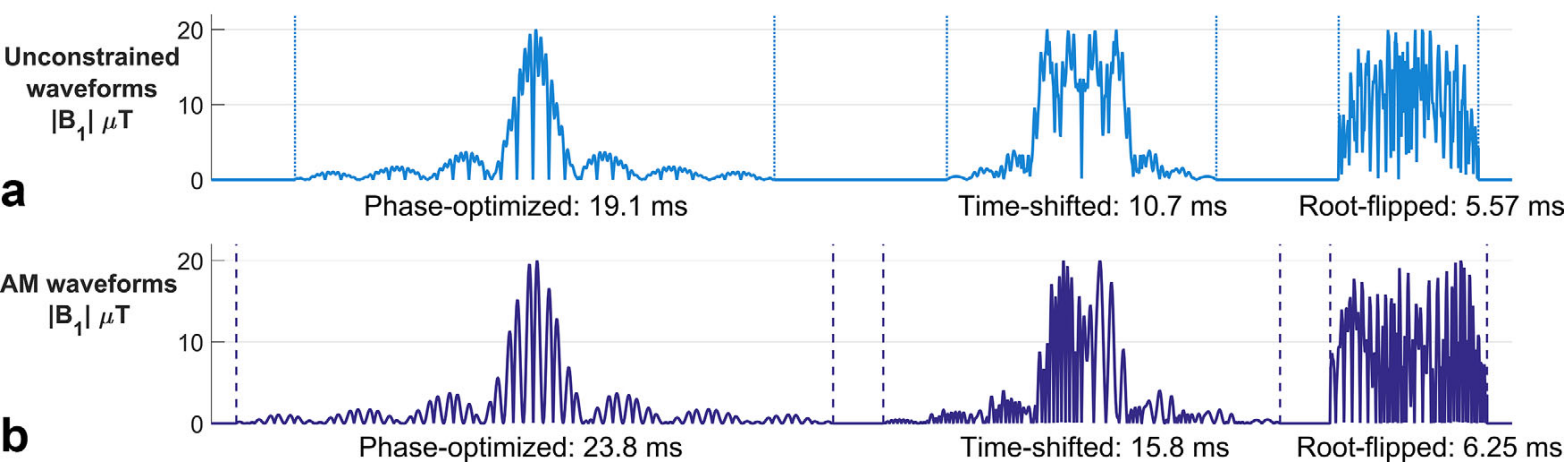
Multiband RF refocusing pulses for multiband factor 4, time‐bandwidth product 6, and slice separation of 5 slices, for each of the three design methods with unconstrained (**a**) and amplitude modulated (**b**) designs. Each pulse has been scaled to 20 μT peak amplitude. The AM condition results in longer pulse durations.

Figure 3 shows sample magnitude waveforms for refocusing pulses with MB = 4, TBP = 6, and slice separation = 5 slice thicknesses, designed using the three techniques with and without the AM constraint.

**Figure 4:**
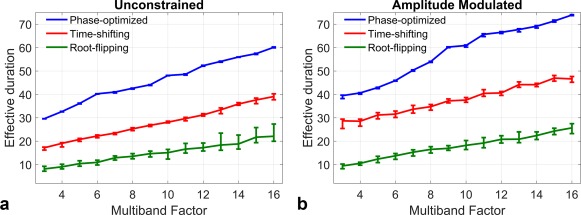
The effective durations of the three techniques in relation to each other, as a function of the multiband factor in the unconstrained (**a**) and AM (**b**) case. All pulses were refocusing pulses designed for a time‐bandwidth product of 6 and averaged over 20 different slice separations. Error bars indicate the maximum and minimum amplitude found in the group of 20 slice separations.

Figure 4 plots the mean $t_{eff}$ averaged over slice separations against multiband factor for each of the unconstrained (4a) and AM (4b) methods at a fixed time‐bandwidth product of 6; error bars indicate the maximum and minimum durations found across the 20 different slice separations tested. Phase‐optimizing is outperformed by time‐shifting, which in turn is outperformed by root‐flipping, as expected. Some variability is observed with respect to slice separation for all methods. This is the case for phase‐optimizing, even though the solutions are independent of slice separation, because the peak of the modulation function is not always directly aligned with the peak of the underlying single‐band waveform. The effect is more pronounced for closely spaced slices.

**Figure 5:**
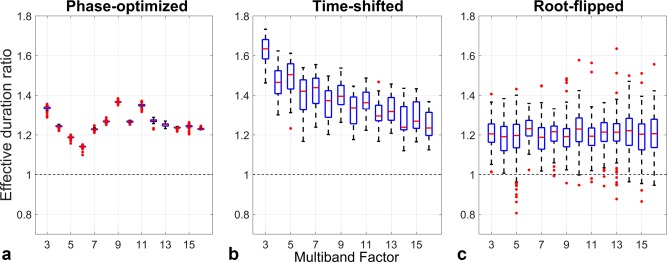
Effective durations of AM multiband pulses relative to their unconstrained design equivalent, for the three techniques. Each AM design is compared with its unconstrained equivalent over all time‐bandwidth products and slice separations (100 different cases for each multiband factor). Phase offsetting shows some variation with the multiband factor, but little variation for different TBP/slice separation, as expected, as this method is independent of the single‐band starting waveforms. Time‐shifting and root‐flipped results show significantly more variation. The AM time‐shifting is comparatively worse at lower multiband factors, whereas root‐flipping is relatively constant across multiband factors. There are some cases in which the AM root‐flipped designs outperform the original method (ie, ratio < 1).

Figure 5 displays the ratio of the effective durations of each AM pulse and its unconstrained counterpart for each method when plotted against multiband factor. The spread of values for each multiband factor is associated both with slice separation and time‐bandwidth product. Averaged across slice separations and time‐bandwidth products, the AM constraint results in effective durations that are longer by 26% for phase‐optimizing, 38% for time‐shifting, and 20% for root‐flipping. Phase‐optimizing results vary insignificantly across the various designs, but there is a significant amount of variance particularly in the latter two methods. Time‐shifting has the largest variation; relative performance of AM‐constrained solutions is particularly poor for time‐shifting at low multiband factors, but improves as this increases. For the phase‐optimization method, multiband factors of 5 (19%) and 6 (14%) are particularly favorable for AM pulses. Average performance for root‐flipped designs is relatively stable across multiband factor and time‐bandwidth product but varies moderately with slice separations. The breakdown of this performance among different time‐bandwidth products can be found in Supporting Figure S2. A notable exception to the general trend occurs for root‐flipped pulses designed for TBP = 2, MB = 5; in this case the AM designs outperform the original method on average.

**Figure 6:**
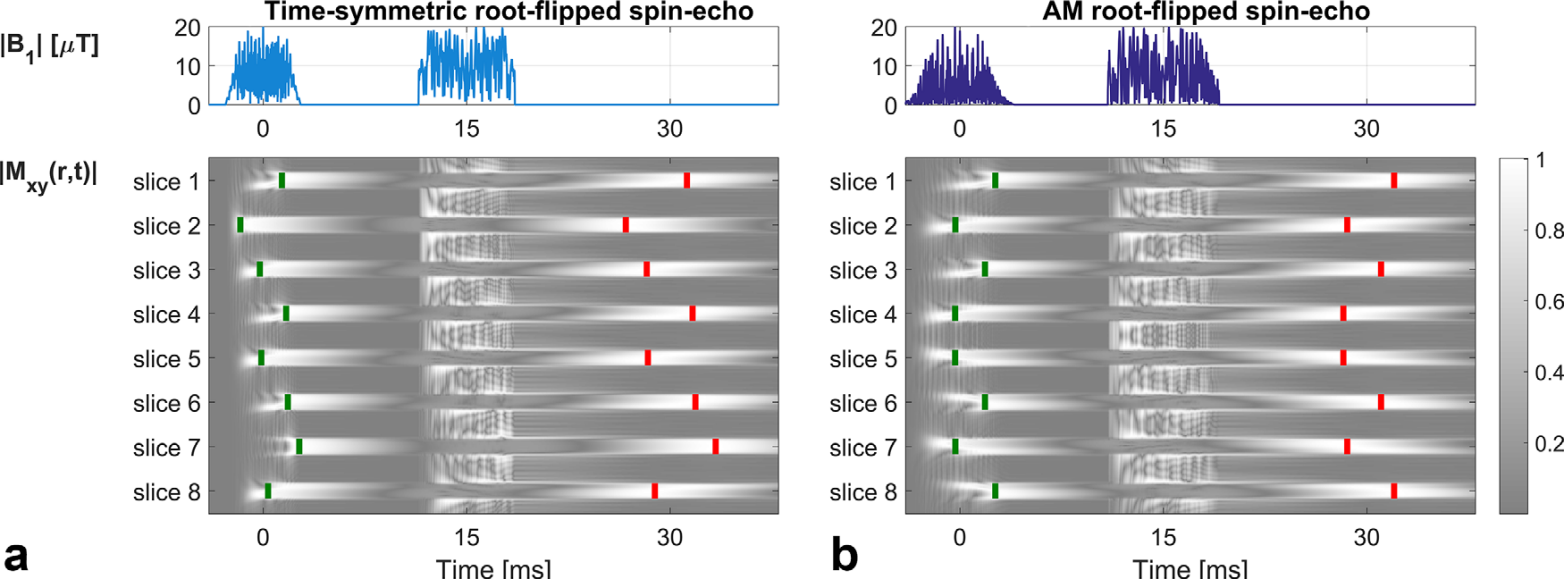
A spin‐echo simulation with a single refocusing pulse for a multiband factor 8 root‐flipped pulse pair of time‐bandwidth product 6, and 3 slice separations. In both the original (time‐symmetric) and AM method, the pulses are scaled to a maximum B_1_ of 20 μT (ie, minimum duration). Green boxes denote the peak M_xy_ during excitation (effective time of flip‐down), and red boxes denote the peak of the refocused echo. Root‐flipped pulses show a spread in both of these times across the group of slices, meaning that each slice will be read out with a different combination of $T_{2}$ and $T_{2}^{*}$ weighting. The effect of conjugate symmetry in the AM case is to make equidistant slices have the same timing.

Sharma et al 5 showed that combining root‐flipped refocusing pulses with minimum‐duration matched excitation pulses leads to a dispersion of echo times. The effect was visualized by averaging transverse magnetization from Bloch simulations for isochromats over a range of frequency offsets ± 50 Hz. Figure 6 shows the result for designs with TBP = 6, MB = 8, and slice separation = 3 slices; the green bars mark the effective time of excitation (peak $\left|M_{xy}\right|$ during excitation) and red bars mark the effective echo times (peak of the refocused $\left|M_{xy}\right|$).

**Figure 7:**
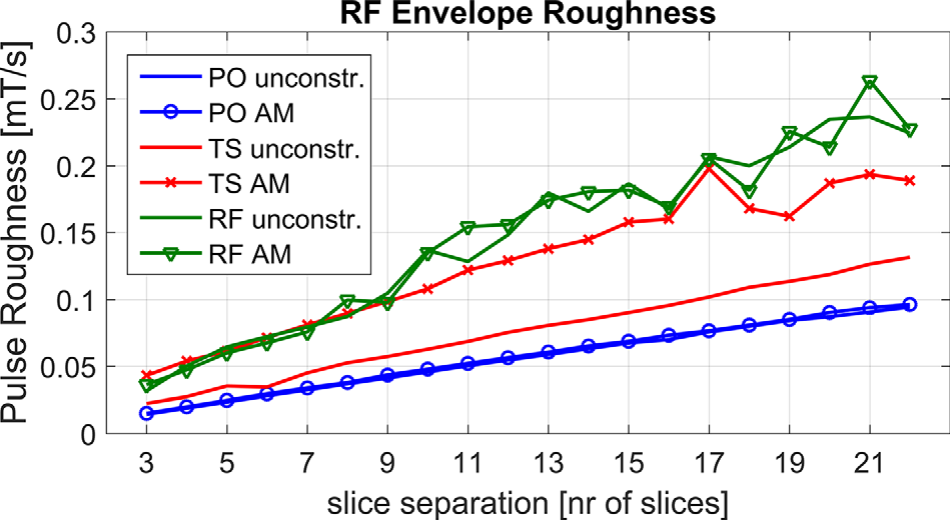
Pulse envelope roughness defined by Equation 12 for the different design cases for MB = 6 TBP = 4. For this analysis, pulses were matched to the same amplitude but varied in duration.

Figure 7 shows the pulse envelope roughness for both unconstrained and AM‐only MB6 TB4 waveforms designed using all three methods. The roughness values differ among the three design methods, with root‐flipped pulses perhaps unsurprisingly giving the highest roughness. For root‐flipped and phase‐optimized methods, there does not appear to be a systematic difference between the AM constrained and unconstrained approaches. However, there is a significant increase in roughness when using the AM constraint for time‐shifted RF pulses.

### Experiments

**Figure 8:**
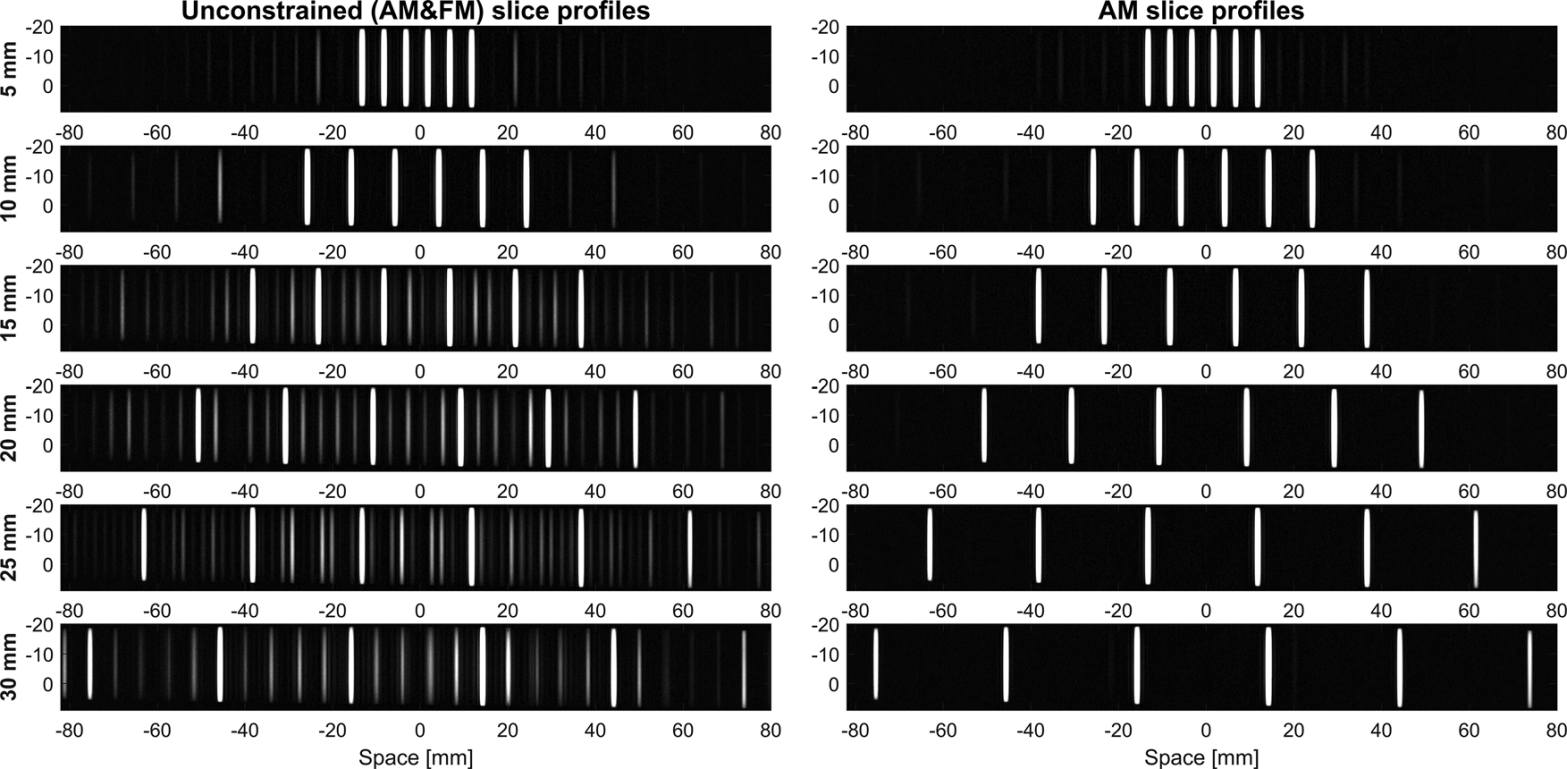
Slice profiles from phantom experiments at 3T with MB = 6, TBP = 2.13 phase‐optimized excitation pulses at different slice separations. All images are windowed in the same way. At low slice separations, the unconstrained waveforms—which are specified using both AM and FM— produce noticeable artifacts that become more significant at higher slice separations. This is not the case for slices excited by AM waveforms, even at high slice separations. All pulses were designed with the same underlying single‐band waveform, and therefore should display the same level of between‐slice ripple; the observed artifacts are interpreted as a pulse generation issue. Sample pulse waveforms are shown in Figure 2.

Figure 8 shows the experimental results from imaging a water phantom at 3T. At low slice separations, the unconstrained waveforms produce small artifacts that become more significant at higher slice separations. Figure 2 demonstrates that increasing the slice gap leads to larger amplitude of the FM waveform. These artifacts are not present when using the AM waveforms, even at high slice separations. The pulses use matched durations and sampling rates that were matched to the system dwell time, to avoid resampling that can lead to additional errors, particularly for FM waveforms.

**Figure 9:**
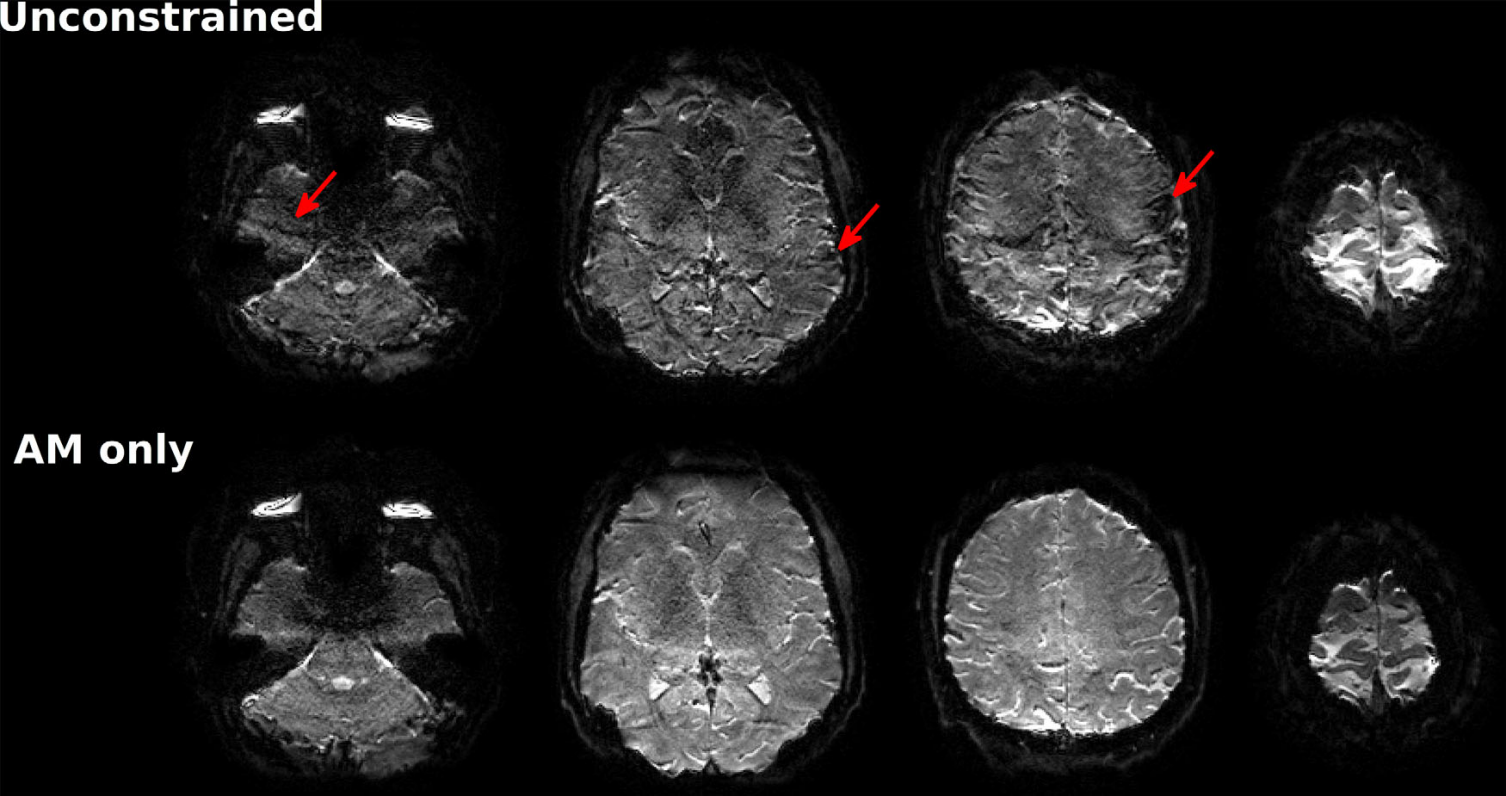
In vivo MB4 GE‐EPI images (1 mm isotropic resolution) using unconstrained and AM‐only phase‐optimized excitation pulses. Images are windowed in the same way. Images from the unconstrained (ie, AM and FM) pulse are affected by incoherent artifacts (arrows) that are attributed to unwanted interslice excitation seen on the slice profile measurements shown in Figure 8. In comparison, the images from the AM‐only pulses are free from this type of artifact. As with the phantom experiment, the RF pulses were matched in design properties and in duration, with the only difference being that the unconstrained pulses were specified using AM and FM, whereas the AM‐only pulses had no FM component.

Results from the in vivo experiments are shown in Figure 9, which compares the unfolded simultaneously acquired (MB4) slices using both the unconstrained and AM‐only RF pulses. Despite the design properties of these pulses being precisely matched, so that in an ideal system they would produce almost identical signals, it is evident that there are additional artifacts in the images from the FM pulse (top row, arrows).

## DISCUSSION

We have presented a methodology for the constrained design of AM‐multiband RF pulses, which have the potential advantage of being less demanding for some types of scanner pulse generator. The motivation for doing so is illustrated by Figure 8, which shows the experimentally measured slice profiles for unconstrained RF pulses (ie, AM and FM modulation) compared with AM‐only RF pulses, which are designed with nominally the same characteristics (eg, TBP, ripple). The unconstrained pulses result in artifacts that are not present when using AM‐only designs, and are not predicted from simulating the slice profiles. The severity of the artifacts gets worse as the slices are moved apart (hence, the degree of frequency modulation increases). The effect of this is to produce imaging artifacts of the type shown in Figure 9. The additional interslice excitation is not unfolded by the reconstruction and produces interference across the field of view. This effect has been consistently observed with the scanner used for these experiments; the prevalence of this issue across other hardware is not known. Emerging fully digital hardware systems could avoid this issue; nevertheless, the AM‐only designs explored in this work provide an alternative that is compatible with systems that suffer from this type of issue.

AM‐constrained designs yield equivalent magnitude slice profiles, but are on average 20 to 38% less efficient than unconstrained solutions, depending on the design method used. This conclusion is based on tests using three design methods (phase‐optimizing 3, time‐shifting 4, root‐flipping 5), which were modified by imposing conjugate symmetry on the relevant frequency domain representation. Results were expressed in terms of the effective pulse duration ( $t_{eff}$) relative to a hard pulse of the same flip angle. This provides a neutral basis for comparing pulses without having to specify RF amplitude or pulse duration settings.

Root‐flipping produced the most efficient RF pulses (shortest $t_{eff}$) for both the AM and unconstrained cases, with time‐shifting second and phase‐optimizing last (Fig. 4). This hierarchy is consistent with the results of Sharma et al 5, and should be expected, as the performance mirrors the number of degrees of freedom available to each method. Sample waveforms from each method (Fig. 3) illustrate this difference, as the root‐flipping allows for the most even distribution of RF energy throughout the duration of the pulse.

Constraining each method to produce purely AM RF pulses generally leads to a loss of performance, as shown in Figure 5. Given the reduction in number of degrees of freedom, the loss of performance is perhaps surprisingly small, particularly for root‐flipping in which the average difference is 20%. Of all of the methods examined, time‐shifting suffers the largest loss in performance, particularly at low multiband factors. We suspect this is because the required constraints (slices must be time‐shifted in pairs and have paired phase offsets) are more limiting for this method.

However, it is striking that there are some design cases in which AM pulses incur very low penalties compared with their conventional counterparts. The phase‐optimization and time‐shifting AM designs are never better than unconstrained designs; indeed this is to be expected, as the AM case is a subset of the general optimization problem. However, AM phase‐optimization performance is particularly good for MB = 5 and 6, in which the duration penalties are only approximately 19 and 14%, respectively. For root‐flipping, there are cases in which the AM designs are better (the relative duration is less than 1). Figure 5 indicates that some AM designs are better than the conventional case, which is true for TBP = 2 and MB = 5 (Supporting Fig. S2). This is possible for the root‐flipping method, because the AM solutions are not a simple subset of the original solutions. In the original root‐flipping method, if a root is flipped on one half of the complex plane, the conjugate root (the mirror root about the real axes) on the bottom half is not flipped, and vice versa, resulting in time‐symmetric RF pulses. In the proposed AM root‐flipping approach, roots must appear symmetrically about the real axis, which means equidistant slices have symmetric root patterns and the RF pulse is not time‐symmetric. Hence, the two are designed using mutually exclusive symmetry constraints on the root‐flipping. This suggests that there may be even more efficient solutions for truly unconstrained root‐flipped pulses, which could merit further investigation.

Time‐shifting and root‐flipping can lead to spin echoes from different slices that are not aligned in time; the precise timing depends on the design of the matched excitation pulse in each case. A consequence of the AM constraint is that, in the case of time‐shifting, the underlying single‐slice waveforms must be shifted in pairs, so these pairs of slices have the same temporal properties. Similarly, we observed that a consequence of the AM root‐flipping patterns when combined with minimum duration excitations is that spin echoes also form in pairs, with slices spaced equally about the center frequency, forming echoes at the same point in time (Fig. 6b). This is in contrast to the original method, which created time‐symmetric pulses resulting in antisymmetric echo times for equidistant slices (Fig. 6a and Fig. 7 in 5). Discrepancies in echo times are an inevitable aspect of multiband excitation/refocusing with these techniques, and characterization of these effects could be an interesting topic for future investigation.

In addition to the hardware issues that have motivated this study, there are other more practical benefits of using AM pulses, which although not the main motivation for this work, could prove to be useful. For example, AM waveforms are typically less susceptible to resampling errors, and calculation of flip angles can be achieved by integrating the waveform directly (for odd‐numbered MB). Our key motivation was to produce a practical work‐around for hardware issues that arise from the strong demands placed on the RF system by multiband designs. Although our work focused on a pulse generation issue, other authors have examined related problems with rapidly modulated RF pulses. Grissom et al 11 found that rough RF pulse envelopes can lead to fidelity errors on systems with simpler RF amplifier designs. Another study 13 found that a specific absorption rate (SAR) monitoring device with low temporal resolution leads to overestimation of SAR, and hence overly conservative operational limits when using multiband pulses. Multiband pulses clearly have much rougher profiles than traditional single‐band pulses; however, Figure 7 suggests that for the root‐flipped and phase‐optimized methods there is no systematic difference in “envelope roughness” between the AM and unconstrained approaches. There is, however, a consistent increase in roughness when using time‐shifted pulses. The AM constraint generally leads to poorer performance with time‐shifted designs, largely because of the requirement that pulses be shifted in pairs. Although the aforementioned power amplifier and SAR monitoring issues were not a concern for the hardware used in this work, additional design constraints on roughness are a possibility for some design approaches 11.

## CONCLUSIONS

Existing multiband pulse design methods can be modified to produce real AM‐only multiband pulse waveforms. The AM‐only waveforms can be realized more reliably on some hardware, as illustrated in this study. These pulses come at a cost in duration, compared with their corresponding unconstrained versions; however, this cost is relatively modest for phase‐optimizing (26%) and root‐flipping (20%), but larger for time‐shifting (38%). The required symmetry constraint also leads to different timing of spin‐echo formation when used with root‐flipping.

## Supporting information

Additional Supporting Information may be found in the online version of this article

**Fig. S1**. The “edge spike” of a minimum‐phase filter can be moved by flipping stop‐band roots. Just as flipping passband roots controls passband energy across the pulse duration, so does flipping stopband roots control the distribution of stopband energy. The top row shows an untouched problematic minimum‐phase filter with all stopband roots outside the unit circle and the spike at the end of the filter in the time domain. This spike (annotated with arrows) will remain, regardless of how the passband roots are flipped. The second row shows that when most of the stopband roots are flipped inside the unit circle, the spike moves to the start. When stopband roots are flipped alternatively, the spike moves to the center. We found that a good solution is to divide the stopband on each half‐circle into subbands (six was found to work well), and flip each band alternatively (ie, like a square waveform). This increases roughness around the pulse edges, without the stopband energy accumulating at any coefficient in particular.
**Fig. S2**. The relative AM performance for the three methods for different time‐bandwidth products, as in Figure 5 but now resolved for different TBP. The general trends follow those in Figure 5 with some exceptions. For example, for TBP = 2, the AM‐constrained root‐flipped pulses are on average better than the unconstrained versions for MB = 5.
