# Supplementary material for: Optimized amplitude modulated multiband RF pulse design
Source: Magn Reson Med. 2017 Jan 17;78(6):2185–93. doi: 10.1002/mrm.26610 (PMC5697703; doi:10.1002/mrm.26610)
Supplement: Supplementary file 1 — Fig. S1. The “edge spike” of a minimum‐phase filter can be moved by flipping stop‐band roots. Just as flipping passband roots controls passband energy across the pulse duration, so does flipping stopband roots control the distribution of stopband energy. The top row shows an untouched problematic minimum‐phase filter with all stopband roots outside the unit circle and the spike at the end of the filter in the time domain. This spike (annotated with arrows) will remain, regardless of how the passband roots are flipped. The second row shows that when most of the stopband roots are flipped inside the unit circle, the spike moves to the start. When stopband roots are flipped alternatively, the spike moves to the center. We found that a good solution is to divide the stopband on each half‐circle into subbands (six was found to work well), and flip each band alternatively (ie, like a square waveform). This increases roughness around the pulse edges, without the stopband energy accumulating at any coefficient in particular. Fig. S2. The relative AM performance for the three methods for different time‐bandwidth products, as in Figure 5 but now resolved for different TBP. The general trends follow those in Figure 5 with some exceptions. For example, for TBP = 2, the AM‐constrained root‐flipped pulses are on average better than the unconstrained versions for MB = 5. [file MRM-78-2185-s001.docx]

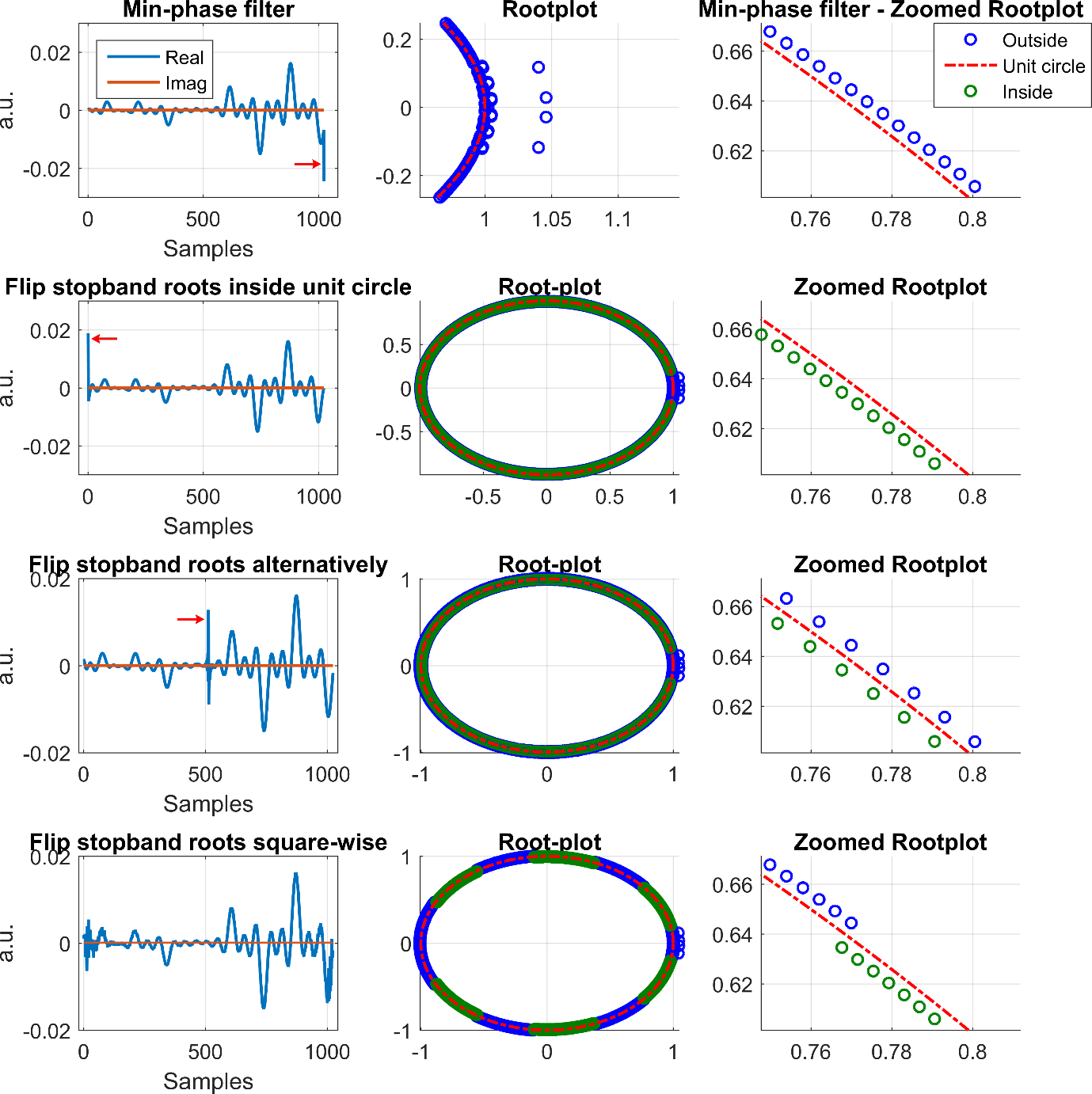


**Supporting Figure S1:** The “edge-spike” of a minimum-phase filter can be moved by flipping stop-band roots. Just as flipping passband roots controls passband energy across the pulse duration, so does flipping stopband roots control the distribution of stopband energy. The top row shows an untouched problematic minimum-phase filter with all stop-band roots outside the unit circle and the spike at the end of the filter in the time-domain. This spike (annotated with arrows) will remain regardless of how the passband roots are flipped. The second row shows that when most of the stopband roots are flipped inside the unit circle, the spike moves to the start. When stop-band roots are flipped alternatively, the spike moves to the center. We found that a good solution is to divide the stop-band on each half-circle into sub-bands (six was found to work well), and flip each band alternatively (i.e. like a square waveform). This increases roughness around the pulse edges, without the stopband energy accumulating at any coefficient in particular.


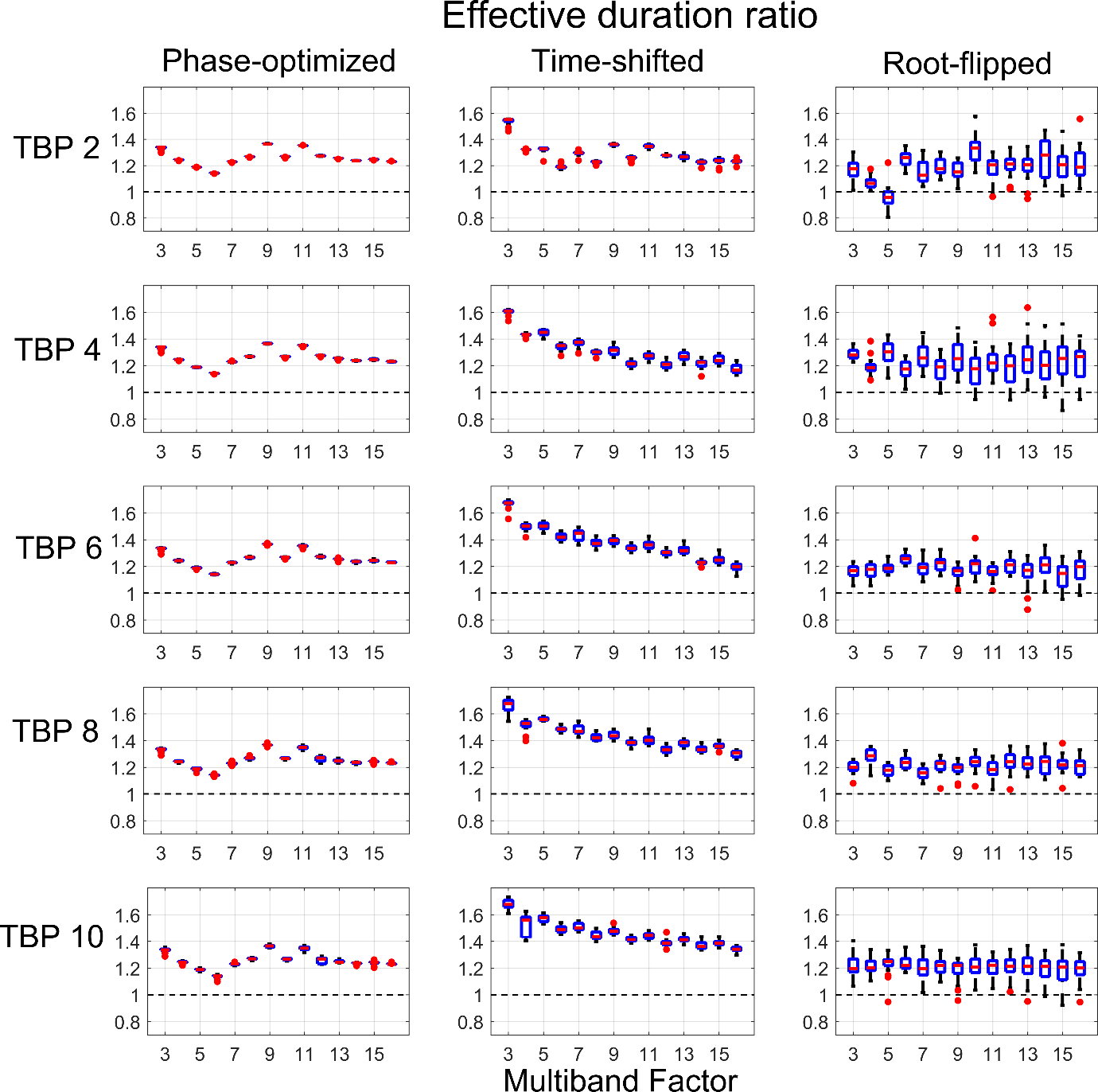


**Supporting Figure S2:** The relative AM performance for the three methods for different time-bandwidth products; as Figure 5 but now resolved for different TBP. The general trends follow those in Figure 5 with some exceptions. For example for TBP=2 the AM constrained root-flipped pulses are on average better than the unconstrained versions for MB=5.
